# Draft Genome Sequence of *Burkholderia pyrrocinia* Lyc2, a Biological Control Strain That Can Suppress Multiple Plant Microbial Pathogens

**DOI:** 10.1128/genomeA.00991-14

**Published:** 2014-10-02

**Authors:** Xiao-Qiang Wang, Kurt C. Showmaker, Xiao-Qing Yu, Tao Bi, Chuan-Yu Hsu, Sonya M. Baird, Daniel G. Peterson, Xiang-Dong Li, Shi-En Lu

**Affiliations:** a Department of Plant Pathology, College of Plant Protection, Shandong Agricultural University, Tai’an, Shandong, China; b Department of Biochemistry, Molecular Biology Entomology and Plant Pathology, Mississippi State University, Mississippi State, Mississippi, USA; c Collaborative Innovation Centre for Annually High Yield and High Efficiency Production of Wheat and Corn, Shandong Agricultural University, Tai’an, Shandong, China; d The Institute for Genomics, Biocomputing & Biotechnology, Mississippi State University, Mississippi State, Mississippi, USA

## Abstract

*Burkholderia pyrrocinia* strain Lyc2 was isolated from the tobacco rhizosphere in China. This bacterium exhibits a remarkable capacity to inhibit the growth of multiple pathogens and shows strong suppression of cotton seedling damping-off. Here, we present the draft genome sequence of *Burkholderia pyrrocinia* strain Lyc2.

## GENOME ANNOUNCEMENT

The *Burkholderia cepacia* complex (BCC) is composed of a group of genetically different but phenotypically similar bacteria (1). A striking feature of some *Burkholderia* strains is their production of various antimicrobial compounds, which show great potential in plant disease management (2–7). However, the use of *Burkholderia* strains as living biocontrol agents has been reconsidered, since some BCC isolates are reported to be opportunistic pathogens associated with the human disease cystic fibrosis (8, 9). BCC strain Lyc2 was isolated from tobacco rhizosphere and identified to be *Burkholderia pyrrocinia* (10). Lyc2 can suppress both fungal and bacterial pathogens of agricultural significance *in vitro*, such as *Rhizoctonia solani*, *Rhizoctonia cerealis*, *Fusarium oxysporum* f. sp. *vasinfectum*, *F. oxysporum* f. sp. *cucumerinum*, *Fusarium moniliforme*, *Colletotrichum gloeosporioides*, *Colletotrichum orbiculare*, *Sclerotinia sclerotiorum*, *Alternaria alternata*, *Clavibacter michiganensis*, and *Erwinia amylovora*. Understanding the genetic elements responsible for antimicrobial production in *Burkholderia* strains will provide important clues to the development of biocontrol chemicals and the elimination of the potential health risks of BCC. Here, we describe the draft genome sequence of strain Lyc2 to better understand the genetic background of Lyc2 as a potential biocontrol agent.

Genomic DNA from Lyc2 was prepared with the cetyltrimethylammonium bromide (CTAB) protocol (11). An Illumina TruSeq DNA PCR-free library was prepared, and sequenced paired ends of 300 bp were prepared with Illumina MiSeq reagent kit version 3 on the MiSeq instrument. The resultant 15.2 million reads were trimmed with Trimmomatic-0.30 and assembled with Velvet (version 1.2.10) (12, 13). The assembly resulted in 74 contigs with a combined length of 7,798,267 bp and an *N*_50_ of 504,913 bp. Two contigs were >1 Mb, while 16 contigs were >100 kb. Electronic annotation by the NCBI Prokaryotic Genome Annotation Pipeline identified 6,643 protein-coding genes and 60 tRNAs (14). The draft assembly G+C content is 66.7%, which is similar (67.37%) to that of the 131 contigs present in the draft assembly of *B. pyrrocinia* CH-67 (15).

BLASTn alignments revealed the presence of gene clusters putatively involved in the synthesis of pyrrolnitrin (16), malleobactin (17), phenazine (18), and AFC-BC11 (19), which play important roles in biological control activity. Interestingly, a 55.2-kb genomic region sharing a significant identity (92.5%) with the *ocf* cluster of *Burkholderia contaminans* strain MS14, which is responsible for occidiofungin production (7, 20), was identified in the genome (21). A more specific mutation analysis of *B. pyrrocinia* strain Lyc2 will be carried out in future research.

### Nucleotide sequence accession number.

The draft genome sequence of *B. pyrrocinia* strain Lyc2 has been deposited at DDBJ/EMBL/GenBank under the accession no. JPWP00000000. The version described in this paper is the first version.
